# A Novel Certificateless Signature Scheme for Smart Objects in the Internet-of-Things

**DOI:** 10.3390/s17051001

**Published:** 2017-05-01

**Authors:** Kuo-Hui Yeh, Chunhua Su, Kim-Kwang Raymond Choo, Wayne Chiu

**Affiliations:** 1 Department of Information Management, National Dong Hwa University, Hualien 97401, Taiwan; khyeh@gms.ndhu.edu.tw (K.-H.Y.); 410235014@gms.ndhu.edu.tw (W.C.); 2 Division of Computer Science, University of Aizu, Aizu-Wakamatsu, Fukushima Pref. 965-8580, Japan; 3 Department of Information Systems and Cyber Security, The University of Texas at San Antonio, San Antonio, TX 78249, USA; raymond.choo@fulbrightmail.org

**Keywords:** certificateless signature, Internet-of-things (IoT), security, sensors

## Abstract

Rapid advances in wireless communications and pervasive computing technologies have resulted in increasing interest and popularity of Internet-of-Things (IoT) architecture, ubiquitously providing intelligence and convenience to our daily life. In IoT-based network environments, smart objects are embedded everywhere as ubiquitous things connected in a pervasive manner. Ensuring security for interactions between these smart things is significantly more important, and a topic of ongoing interest. In this paper, we present a certificateless signature scheme for smart objects in IoT-based pervasive computing environments. We evaluate the utility of the proposed scheme in IoT-oriented testbeds, i.e., Arduino Uno and Raspberry PI 2. Experiment results present the practicability of the proposed scheme. Moreover, we revisit the scheme of Wang et al. (2015) and revealed that a malicious super type I adversary can easily forge a legitimate signature to cheat any receiver as he/she wishes in the scheme. The superiority of the proposed certificateless signature scheme over relevant studies is demonstrated in terms of the summarized security and performance comparisons.

## 1. Introduction

The boosting advances on wireless communication and sensing technologies bring universal Internet connectivity, and a more ubiquitous and pervasive computing environment is thus created, called Internet-of-Things (i.e., IoT). Plenty of novel smart objects with specific purposes emerge in IoT to support various innovative applications providing higher intelligence and more convenience to our daily life. Since IoT has attracted significant attention as a key step in furthering intelligent human life in the future, IoT is definitely one of the most promising network paradigms in this computer generation. In an IoT environment, numerous smart objects, such as customized sensors or wearable intelligent devices, can be used to sense, collect, transmit, disseminate, etc., data from the field to a server or other smart things. Unsurprisingly, IoT has wide industrial and individual applications. However, due to the amount and nature of data and potential for exploitation, it is essential to ensure the security of both data-in-transit and data-at-rest [1,2,3,4]. In addition, the heterogeneous nature of the IoT network and the presence of (a large number of) specific-purpose sensors embedded within the smart objects complicate efforts to offer effective security. One particular research challenge is to balance the tradeoff between performance efficiency and system security when designing security solutions for smart objects in IoT-based networks.

In the literature, researchers have dedicated significant efforts on refining traditional security techniques as system security solutions for IoT-based network architectures, such as authentication [5,6,7,8,9], signcryption [10,11,12,13], and certificateless digital signature [14,15], respectively. First of all, due to the nature of limited processing capability of smart objects, the design of lightweight authentication has been thoroughly investigated as a critical security component in IoT-based network systems. In this category of study, lightweight but robust crypto-modules, such as one way hash function, are embedded into the operation and communication of resource-constrained IoT-based objects to support the security of application operated by objects and backend servers (from service providers). It simultaneously focuses on the computation efficiency and communication robustness of object-to-object and object-to-server data exchange procedures. Secondly, the signcryption technique combines the merits from encryption and digital signature. Most of critical security requirements, such as confidentiality, integrity, unforgeability, and non-repudiation, can be guaranteed in a single logic step. It enjoys better security robustness than other kinds of single-crypto-based security mechanisms. Thirdly, the refinement of certificateless digital signature for protecting IoT-based networks has been studied because of the benefit from the relief on the difficult certificate management in traditional public key infrastructure. Relying on a trusted third party, certificateless public key cryptography facilitates users in establishing a private key and the corresponding public key. It is, thus, more suitable to IoT-based network architecture since there is no need to maintain a centralized server for key/certificate management. In addition, with the decentralized and changed structure, it is believed that we the more efficiency will be guaranteed due to the less of limitation on implementing security mechanism on IoT. Existing certificateless signature schemes can be broadly categorized into certificateless signature schemes with and without bilinear pairing. It has been proven that bilinear pairing is less efficient than ECC (elliptic curve cryptography) point-based crypto-operations, in terms of computation costs [16], although the use of bilinear pairing results in shorter signature message. The latter property makes bilinear pairing-based approach particularly suitable for bandwidth-limited networks, such as traditional wireless sensor networks. Nevertheless, owing to the recent advancements in communication technologies, including those for sensors, the communication environment for existing IoT-based sensors is not as limited by bandwidth restriction as before. Various techniques, such as Bluetooth Low Energy, LoRa, and Zigbee, have been leveraged to build IoT-based communication networks which are bandwidth-guaranteed during sensors-to-server message transmission. Hence, during the design of an efficient and secure certificateless signature scheme for IoT-based smart objects, we argue that computation efficiency takes priority over communication efficiency. For the above observations, in this paper we focus on the design of a certificateless signature scheme with ECC point-based crypto-operations for IoT-based network environments.

The rest of the paper is organized as follows. Section 2 presents relevant background materials. In Section 3, we present the proposed certificateless signature scheme for IoT-based smart objects. We then provide the security analysis and the system implementation of our proposed scheme in Section 4 and Section 5, respectively. In Section 6, we review related work and present a comparative summary, in terms of security and performance. Finally, we conclude the paper in Section 7.

## 2. Preliminary

The objective of this study is to propose a robust and efficient certificateless signature scheme with ECC point-based crypto-operations. ECC is one kind of public key cryptography (PKC)-based techniques, where it is based on the algebraic structure of elliptic curves over finite fields. Normally, ECC requires a smaller key size than other PKC-oriented approaches to provide an equivalent security level. For example, it is generally thought that the same security can be delivered by 256-bit elliptic curve and 3072-bit RSA. Hence, to enjoy higher computation efficiency, we would like to integrate the ECC crypto-technique into our proposed certificateless signature scheme. Furthermore, since the robustness of the proposed scheme is based on the hardness of solving the Elliptic Curve Discrete Logarithm Problem (ECDLP), we present the definition of ECDLP in the following.

- The ECDLP is defined as follows: Let the notation $E/E_{p}$ denotes an elliptic curve E over a prime finite field $E_{p}$, defined by an equation: $y^{2}=x^{3}+ax+b$, where $a,\;b\in F_{p}$ are constants such that $\Delta =4a^{3}+27b^{2}\neq 0$. All points *P_i_* = (*x_i_*, *y_i_*) on *E* and the infinity point *O* form a cyclic group *G* under the operation of point addition *R* = *P* + *Q* defined based on the chord-and-tangent rule. In addition, *t* · *P* = *P* + *P* + … + *P* (*t* times) is defined as a scalar multiplication, where *P* is a generator of *G* with order *n*. The ECDLP is that given a group *G* of elliptic curve points with prime order *n*, a generator *P* of *G* and a point *x* · *P*, it is computationally infeasible to derive *x*, where $x\in Z_{n}^{*}$.

**Figure 1:**
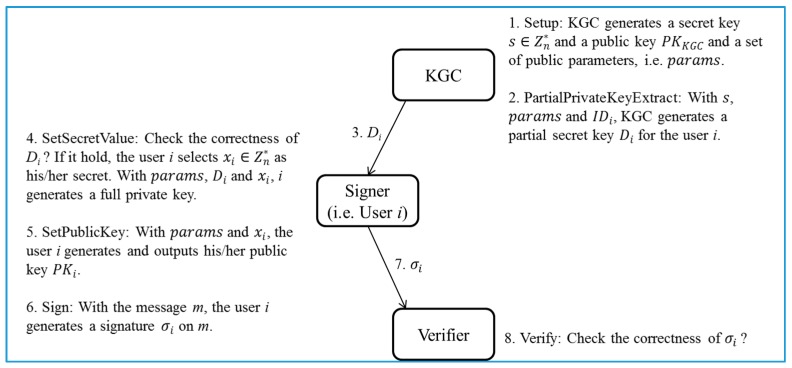
The normal process of a general certificateless signature scheme.

The robustness of the proposed certificateless signature scheme is based on the intractability of ECDLP. Next, for better understanding of our proposed scheme, we present the general concepts of the certificateless signature. A certificateless signature scheme generally consists of six phases, i.e., Setup, PartialPrivateKeyExtract, SetSecretValue, SetPublicKey, Sign, and Verify [17]. Note that the four phases, i.e., Setup, PartialPrivateKeyExtract, SetSecretValue, and SetPublicKey, can be treated as a pre-processing stage. In the following, we briefly review the normal process of a general certificateless signature scheme (Figure 1).

- Step 1 (Setup phase): A trusted KGC (key generation center) generates a master secret key $s\in Z_{n}^{*}$, a corresponding master public key $PK_{KGC}$ and a set of public parameters, i.e., params.
- Step 2 (PartialPrivateKeyExtract phase): With the master secret key s, params and the user *i*’s identity $ID_{i}$, KGC generates a partial secret key $D_{i}$ for the user *i*.
- Step 3: KGC sends $D_{i}$ to the user *i*.
- Step 4 (SetSecretValue phase): Upon receiving $D_{i}$, the user *i* examine the correctness of $D_{i}$. If it holds, the user *i* randomly selects a value $x_{i}\in Z_{n}^{*}$ as his/her secret. Otherwise, the session is terminated.
- Step 5 (SetPublicKey phase): With params and $x_{i}$, the user *i* generates and outputs his/her public key $PK_{i}$.
- Step 6 (Sign phase): With the message *m*, this phase outputs a signature $\sigma_{i}$ which is based on *m*, s and $x_{i}$.
- Step 7: the user *i* sends $\sigma_{i}$ to the verifier.
- Step 8 (Verify phase): With the signature $\sigma_{i}$ of the message *m*, the verifier examine the correctness of $\sigma_{i}$. If the examination holds, the signature is valid. Otherwise, the session is terminated.

## 3. The Proposed Certificateless Signature Scheme for IoT-Based Smart Objects

In this section, we propose a new certificateless signature scheme with ECC point-based crypto-operations. The security of the scheme assumes the intractability of ECDLP. In the following, we present the proposed scheme consisting of two phases, i.e., the Pre-processing phase and Sign/Verify phase. Note that three entities, i.e., KGC, the signer and the verifier, are involved.

**Figure 2:**
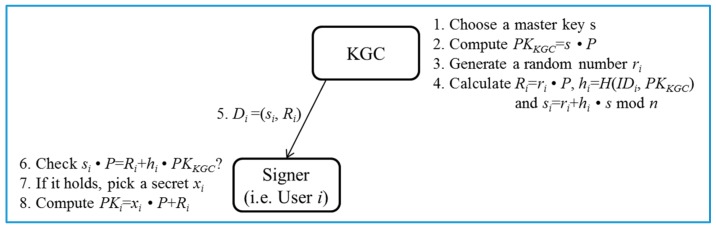
Pre-processing phase of the proposed certificateless signature scheme.

**Figure 3:**
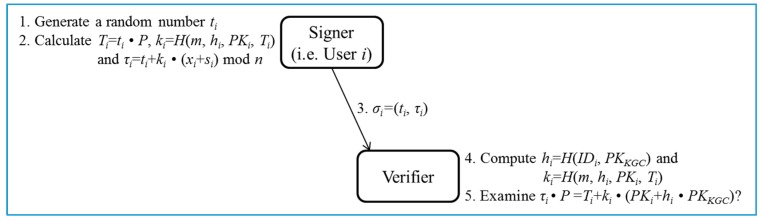
Sign/Verify phase of the proposed certificateless signature scheme.

- Pre-processing phase (Figure 2):
  ○ Steps 1–4: KGC generates a group *G* of elliptic curve points with prime order *n* and determines a generator *P* of *G*. Then, KGC chooses a master key $s\in Z_{n}^{*}$ and a secure hash function $H_{1}:{\left\{0,1\right\}}^{*}\times G\to Z_{q}^{*}$. Next, KGC calculates a master public key $PK_{KGC}=s\cdot P$. Eventually, KGC publishes $params=\left(G,P,PK_{KGC},H\right)$ and keeps s securely. Next, given params, s and the identity $ID_{i}$ of user *i*, KGC generates a random number $r_{i}\in Z_{n}^{*}$, and calculates $R_{i}=r_{i}\cdot P$, $h_{i}=H\left(ID_{i},PK_{KGC}\right)$ and $s_{i}=r_{i}+h_{i}\cdot s$ mod *n*.
  ○ Steps 5–6: KGC returns a partial private key $D_{i}=\left(s_{i},R_{i}\right)$ to the user *i* who checks the validity of $D_{i}$ via whether the equation $s_{i}\cdot P=R_{i}+h_{i}\cdot PK_{KGC}$ mod *n* holds or not. The correctness of $D_{i}$ is presented as follows:
    $$s_{i}\cdot P=\left(r_{i}+h_{i}\cdot s\right)\cdot P=r_{i}\cdot P+h_{i}\cdot s\cdot P=R_{i}+h_{i}\cdot PK_{KGC}.$$
  ○ Steps 7–8: If it holds, the user *i* picks a random number $x_{i}\in Z_{n}^{*}$ as his/her own secret value. Otherwise, the session is terminated. Then, given params and $x_{i}$, the user *i* computes $PK_{i}=x_{i}\cdot P+R_{i}$ as his/her public key.
- Sign/Verify phase (Figure 3):
  ○ Steps 1–3 (Sign): Given params, $D_{i}$, $x_{i}$ and a message *m*, the user *i* first chooses a random number $t_{i}\in Z_{n}^{*}$. Then, the user *i* computes $T_{i}=t_{i}\cdot P$, $k_{i}=H\left(m,h_{i},PK_{i},T_{i}\right)$ and $\tau_{i}=t_{i}+k_{i}\cdot (x_{i}+s_{i})$ mod *n*. Note that the computation of $h_{i}$ is performed at the Pre-processing phase and thus the cost can be removed. Finally, the user *i* outputs $\sigma_{i}=\left(T_{i},\tau_{i}\right)$ as the signature of the message *m*.
  ○ Steps 4–5 (Verify): Given params, $ID_{i}$, $PK_{i}$, and $\sigma_{i}=\left(T_{i},\tau_{i}\right)$, the verifier first computes $h_{i}=H\left(ID_{i},PK_{KGC}\right)$ and $k_{i}=H\left(m,h_{i},PK_{i},T_{i}\right)$. Next, the verifier examines if $\tau_{i}\cdot P=T_{i}+k_{i}\cdot \left(PK_{i}+h_{i}\cdot PK_{KGC}\right)$ holds. The signature $\sigma_{i}$ is accepted if the equation holds. The correctness of the signature $\sigma_{i}=\left(T_{i},\tau_{i}\right)$ is presented as follows:
    $$\tau_{i}\cdot P=\left(t_{i}+k_{i}\cdot (x_{i}+s_{i}\right))\cdot P$$
    $$=t_{i}\cdot P+k_{i}\cdot (x_{i}+r_{i}+h_{i}\cdot s)\cdot P$$
    $$=T_{i}+k_{i}\cdot \left(x_{i}\cdot P+r_{i}\cdot P+h_{i}\cdot s\cdot P\right)$$
    $$=T_{i}+k_{i}\cdot \left(\left(x_{i}\cdot P+R_{i}\right)+h_{i}\cdot PK_{KGC}\right)$$
    $$=T_{i}+k_{i}\cdot \left(PK_{i}+h_{i}\cdot PK_{KGC}\right)$$

## 4. Security Analysis

We will now define the adversary model we used to prove the security of our scheme, prior to presenting the security analysis.

### 4.1. Adversary Model for Certificateless Signature

In the proposed certificateless signature scheme, we considered type I adversary and type II adversaries as defined in [18]. Due to the lack of certificate verification, it is possible for adversaries to replace an entity's public key with one of its choice. Therefore, the type I adversary models an external adversary capable of replacing any entity’s public key with specific values chosen by the adversary itself. Nevertheless, the type I adversary does not know the private key of KGC. On the other hand, the type II adversary models a malicious KGC who is able to access the master key, but cannot replace the public keys of other entities. In addition, type I and II adversaries can be further classified into three categories of power levels [17,19], i.e., normal adversary, strong adversary, and super adversary. A normal-level adversary only has the ability to learn a valid verification message. A strong-level adversary is able to replace a public key in order to forge a valid verification message when the adversary possesses a corresponding private value. A super-level adversary is able to learn valid verification messages for a replaced public key without any submission. Normally, the super adversary may issue the following queries.

- $CreateUser\left(ID_{t}\right)$: The oracle takes as input a query $\left(ID_{t}\right)$, where $ID_{t}$ is the party t’s identity, and then runs algorithms PartialPrivateKeyExtract, SetSecretValue, and SetPublicKey to obtain the partial private key $D_{t}$, the secret value $x_{t}$, and the public key $PK_{t}$.
- $RequestPublicKey\left(ID_{t}\right)$: The oracle takes as input a query $\left(ID_{t}\right)$. It browses the list L and returns the party t’s public key $PK_{t}$.
- $ReplacePublicKey\left(ID_{t},PK_{t},PK_{t}^{'}\right)$: The oracle takes as input a query $\left(ID_{t},PK_{t},PK_{t}^{'}\right)$. This oracle replaces the party t’s public key with $PK_{t}^{'}$ and updates the corresponding information in the list L.
- $ExtractSecret\left(ID_{t}\right)$: The oracle takes as input a query $ID_{t}$. It browses the list L and returns the secret values $x_{t}$. However, if the party t has been asked the ReplacePublicKey query, it returns ⊥.
- $ExtractPartialSecret\left(ID_{t}\right)$: The oracle takes as input a query $ID_{t}$. It then browses the list *L* and returns the partial private key $D_{t}=\left(s_{t},R_{t}\right)$.
- $SuperSign\left(ID_{t},m_{t}\right)$: The oracle takes as input a query $\left(ID_{t},m_{t}\right)$, where $m_{t}$ denotes the message to be signed. This oracle outputs a signature $\sigma_{t}=\left(R_{t},T_{t},\tau_{t}\right)$ such that $true\leftarrow Verify\left(m_{t},\sigma_{t},params,ID_{t},PK_{t}\right)$. If the public key has not been replaced, i.e., $PK_{t}=PK_{t}$, $PK_{t}$ is the public key returned from the oracle $RequestPublicKey\left(ID_{t}\right)$. Otherwise, $PK_{t}=PK_{t}^{'}$, where $PK_{t}^{'}$ is the latest public key value submitted to the oracle $ReplacePublicKey\left(ID_{t},PK_{t},PK_{t}^{'}\right)$.

The following two games, i.e., Games 1 and 2, are against super type I and type II adversaries, respectively. Type I adversary models an external adversary who is able to replace any entity’s public key with specific values chosen by the adversary itself. On the other hand, type II adversary simulates a malicious KGC who holds the master key and might engage in adversarial activities, such as eavesdropping on signatures and asking signing queries.

**Game** **1.** *This game is performed between a challenger C and a super type I adversary* $SA_{1}$ *interacting within the proposed certificateless signature scheme. First, in the “Initialization” stage, the challenger C runs the Setup algorithm and generates a private key* s, *and public system parameters* params. *Next, C keeps* s, *but gives* params *to the adversary* $SA_{1}$. *Second, in the “Query” phase*, $SA_{1}$ *can adaptively access oracle queries* $CreateUser\left(ID_{t}\right)$, $RequestPublicKey\left(ID_{t}\right)$, $ReplacePublicKey\left(ID_{t},PK_{t},PK_{t}^{'}\right)$, $ExtractSecret\left(ID_{t}\right)$, $ExtractPartialSecret\left(ID_{t}\right)$ *and* $SuperSign\left(ID_{t},m_{t}\right)$, *of C, where t may be the user i. After all necessary queries have been asked*, $SA_{1}$ *outputs a forged signature* $\left(ID_{t},m_{t},\sigma_{t}\right)$. $SA_{1}$ *wins in Game 1 if the following three conditions hold*:

(1) $SA_{1}$ *has never queried the oracle* $ExtractPartialSecret\left(ID_{t}\right)$.
(2) $SA_{1}$ *has never queried the oracle* $SuperSign\left(ID_{t},m_{t}\right)$.
(3) *true* ← *Verify*(*m_t_*, *σ_t_*, *params*, *ID_t_*, *PK_t_*) *where* *PK_t_* *is the current public key of party t and it may be replaced by SA_1_*.

**Definition** **1.** *The proposed certificateless signature scheme is existentially unforgeable against a super type I adversary* $SA_{1}$, *if* $SA_{1}$ *runs in polynomial time* pt, *makes at most* $q_{H}$ *queries to the oracle* Hash(.), $q_{CU}$ *queries to the oracle* $CreateUser\left(ID_{t}\right)$, $q_{EPS}$ *queries to the oracle* $ExtractPartialSecret\left(ID_{t}\right)$, $q_{ES}$ *queries to the oracle* $ExtractSecret\left(ID_{t}\right)$, $q_{PK}$ *queries to the oracle* $RequestPublicKey\left(ID_{t}\right)$, $q_{RPK}$ *queries to the oracle* $ReplacePublicKey\left(ID_{t},PK_{t},PK_{t}^{'}\right)$ *and* $q_{SS}$ *queries to the oracle* $SuperSign\left(ID_{t},m_{t}\right)$ *and* $Succ_{SA_{1}}$ *is negligible, where* $Succ_{SA_{1}}$ *is the success probability that* $SA_{1}$*wins in Game 1.*

**Game** **2.** *This game is performed between a challenger C and a super type II adversary* $SA_{2}$ *interacting within the proposed certificateless signature scheme. First, in the “Initialization” phase, the challenger C runs the Setup algorithm and generates a private key* s, *and public system parameters* params. *Then, C keeps* s, *but gives* params *to the adversary* $SA_{2}$. *Second, in the “Query” phase*, $SA_{2}$ *can adaptively access the oracle queries* $CreateUser\left(ID_{t}\right)$, $RequestPublicKey\left(ID_{t}\right)$, $ReplacePublicKey\left(ID_{t},PK_{t},PK_{t}^{'}\right)$, $ExtractSecret\left(ID_{t}\right)$, $ExtractPartialSecret\left(ID_{t}\right)$ *and* $SuperSign\left(ID_{t},m_{t}\right)$, *of C, where t may be the user i. After all necessary queries have been asked*, $SA_{2}$ *outputs a forged signature* $\left(ID_{t},m_{t},\sigma_{t}\right)$. $SA_{2}$ *wins in Game 2 if the following three conditions hold:*

(1) $SA_{2}$ *has never queried the oracle* $ExtractSecret\left(ID_{t}\right)$.
(2) $SA_{2}$ *has never queried the oracle* $SuperSign\left(ID_{t},m_{t}\right)$.
(3) *true* ← *Verify*(*m_t_*, *σ_t_*, *params*, *ID_t_*, *PK_t_*), *where* $PK_{t}$ *is the original public key of party*.

**Definition** **2.** *The proposed certificateless signature scheme is existentially unforgeable against a super type II adversary* $SA_{2}$, *if* $SA_{2}$ *runs in polynomial time* pt, *makes at most* $q_{H}$ *queries to the oracle* Hash(.), $q_{CU}$ *queries to the oracle* $CreateUser\left(ID_{t}\right)$, $q_{EPS}$ *queries to the oracle* $ExtractPartialSecret\left(ID_{t}\right)$, $q_{ES}$ *queries to the oracle* $ExtractSecret\left(ID_{t}\right)$, $q_{PK}$ *queries to the oracle* $RequestPublicKey\left(ID_{t}\right)$, $q_{RPK}$ *queries to the oracle* $ReplacePublicKey\left(ID_{t},PK_{t},PK_{t}^{'}\right)$ *and* $q_{SS}$ *queries to the oracle* $SuperSign\left(ID_{t},m_{t}\right)$ *and* $Succ_{SA_{2}}$ *is negligible, where* $Succ_{SA_{2}}$ *is the success probability that* $SA_{2}$ *wins in Game 2*.

### 4.2. Formal Analysis

Assuming the hardness of solving ECDLP, we prove that our proposed scheme is existentially unforgeable against the super type I adversary and super type II adversary, respectively.

**Theorem** **1.** *The proposed certificateless signature scheme is existentially unforgeable against a super type I adversary in the random oracle model, assuming the hardness of solving ECDLP. That is, if there exists a super type I adversary* $SA_{1}$ *who can submit queries to random oracles and win in Game 1 with probability* $Succ_{SA_{1}}$, *then there is an algorithm* β *which can solve a random instance of ECDLP in polynomial time with success probability* $Succ_{\beta }\geq \frac{1}{q_{CU}+q_{H}}{\left(1-\frac{1}{q_{CU}+q_{H}}\right)}^{q_{EPS}}Succ_{SA_{1}}$.

**Proof**.  Let $SA_{1}$ be a super type I adversary $SA_{1}$ which can compromise our proposed certificateless signature scheme with a non-negligible probability $Succ_{SA_{1}}$. We then construct a polynomial-time algorithm β which can utilize $SA_{1}$ to solve ECDLP. At first, β contains a hash list $L_{H_{1}}$ and a key list $L_{K_{1}}$, which are initially empty.

- Initialization phase: β picks an identity $ID^{*}$ as the challenged identity in Game 1, sets $PK_{KGC}$ and sends $params=\left(G,P,PK_{KGC},H\right)$ to $SA_{1}$.
- Query phase:
  ➢ $CreateUser\left(ID_{t}\right)$: The oracle takes as input a query $\left(ID_{t}\right)$. If $ID_{t}$ has been created, nothing happens. Otherwise, β runs algorithms PartialPrivateKeyExtract, SetSecretValue, and SetPublicKey to obtain the partial private key $D_{t}$, the secret value $x_{t}$ and the public key $PK_{t}$. Next, β returns $PK_{t}$ to $SA_{1}$.
  ➢ Hash query:
    (1) When $SA_{1}$ accesses a hash query on $\left(ID_{t},PK_{KGC}\right)$, if the list $L_{H_{1}}$ contains $<h_{t},ID_{t},PK_{KGC}>$, β returns $h_{t}$ to $SA_{1}$. Otherwise, β picks a random number $h_{t}\in Z_{n}^{*}$, returns $h_{t}$ to $SA_{1}$, and adds $<h_{t},ID_{t},PK_{KGC}>$ to $L_{H_{1}}$.
    (2) When $SA_{1}$ accesses a hash query on $\left(m,h_{t},PK_{t},T_{t}\right)$, if the list $L_{H_{1}}$ contains $<k_{t},m,h_{t},PK_{t},T_{t}>$, β returns $k_{t}$ to $SA_{1}$. Otherwise, β picks a random number $k_{t}\in Z_{n}^{*}$, returns $k_{t}$ to $SA_{1}$, and adds $<k_{t},m,h_{t},PK_{t},T_{t}>$ to $L_{H_{1}}$.
  ➢ $RequestPublicKey\left(ID_{t}\right)$: Upon receiving a RequestPublicKey query with an identity $ID_{t}$ from $SA_{1}$, β performs the following steps.
    (1) If $ID_{t}\neq ID^{*}$, β selects three random numbers $a_{t},b_{t},x_{t}\in Z_{n}^{*}$, and performs $s_{t}\leftarrow a_{t}$, $h_{t}\leftarrow b_{t}$, $R_{t}\leftarrow a_{t}\cdot P-b_{t}\cdot PK_{KGC}$, and $PK_{t}=x_{t}\cdot P+R_{t}$. Then, β adds $\langle ID_{t},R_{t},h_{t}\rangle$ to list $L_{H_{1}}$, and $\langle ID_{t},s_{t},R_{t}\rangle$ and $\langle ID_{t},PK_{t},x_{t}\rangle$ to list $L_{K_{1}}$, respectively. Finally, β returns $PK_{t}$ to $SA_{1}$.
    (2) Otherwise, β generates three random numbers $a_{t},b_{t},x_{t}\in Z_{n}^{*}$, and sets $R_{t}\leftarrow a_{t}\cdot P$, $h_{t}\leftarrow b_{t}$, $s_{t}\leftarrow ⊥$ and $PK_{t}=x_{t}\cdot P+R_{t}$. Then, β adds $\langle ID_{t},R_{t},h_{t}\rangle$ to list $L_{H_{1}}$, and $\langle ID_{t},⊥,R_{t}\rangle$ and $\langle ID_{t},PK_{t},x_{t}\rangle$ to list $L_{K_{1}}$, respectively. Finally, β returns $PK_{t}$ to $SA_{1}$.
  ➢ $ExtractPartialSecret\left(ID_{t}\right)$: Upon receiving an ExtractPartialSecret query for an identity $ID_{t}$ from $SA_{1}$, β performs the following steps.
    (1) If $ID_{t}=ID^{*}$, β stops the session.
    (2) Otherwise, β looks at $L_{H_{1}}$ for $<ID_{t},s_{t},R_{t}>$. If there exists a record of such a tuple, β returns $s_{t}$ to $SA_{1}$; otherwise, β makes a RequestPublicKey query with $ID_{t}$ and returns $s_{t}$ to $SA_{1}$ accordingly.
  ➢ $ExtractSecret\left(ID_{t}\right)$: When β receives an ExtractSecret query for an identity $ID_{t}$ from $SA_{1}$, β looks for $\langle ID_{t},PK_{t},x_{t}\rangle$ in the list $L_{K_{1}}$. If there is such a tuple, β returns $x_{t}$ to $SA_{1}$. Otherwise, β makes a $ExtractPartialSecret\left(ID_{t}\right)$ query and returns $x_{t}$ to $SA_{1}$.
  ➢ $ReplacePublicKey\left(ID_{t},PK_{t},PK_{t}^{'}\right)$: Once β receives a query for some $\left(ID_{t},PK_{t},PK_{t}^{'}\right)$ from $SA_{1}$, β looks for $\langle ID_{t},PK_{t},x_{t}\rangle$in the list $L_{K_{1}}$. If there exists such a record, β sets $PK_{t}=PK_{t}^{'}$ and $x_{t}=⊥$. Otherwise, β makes a RequestPublicKey query with $ID_{t}$ and then sets $PK_{t}=PK_{t}^{'}$ and $x_{t}=⊥$.
  ➢ $SuperSign\left(ID_{t},m_{t}\right)$: Upon receiving a SuperSign query with $\left(ID_{t},m_{t}\right)$ from $SA_{1}$, β looks for $<ID_{t},s_{t},R_{t}>$ and $\langle ID_{t},PK_{t},x_{t}\rangle$ in the lists $L_{K_{1}}$. Next, β generates a random number $c_{t}\in Z_{n}^{*}$, and computes $\tau_{t}\leftarrow c_{t}$ and $T_{t}=\tau_{t}\cdot P-k_{t}\cdot \left(PK_{t}+h_{t}\cdot PK_{KGC}\right)$. After that, β returns $\sigma_{t}=\left(T_{t},\tau_{t}\right)$ to $SA_{1}$.

Finally, $SA_{1}$ outputs a forged but valid signature $\left(ID_{t},m_{t},\sigma_{t}\right)$. If $ID_{t}=ID^{*}$, β terminates the simulation. Otherwise, β looks for $<h_{t},ID_{t},PK_{KGC}>$, $<k_{t},m,h_{t},PK_{t},T_{t}>$, $\langle ID_{t},s_{t},R_{t}\rangle$, and $\langle ID_{t},PK_{t},x_{t}\rangle$ in the lists $L_{H_{1}}$ and $L_{K_{1}}$. On the other hand, based on the forking lemma [20], if we have the polynomial replay of β with the same random tape and different choices of hash oracle, $SA_{1}$ is able to output another two valid signatures. Eventually, we will have three valid signatures, $i.e.,{\sigma_{t}}^{\left(j\right)}=\left({T_{t}}^{\left(j\right)},{\tau_{t}}^{\left(j\right)}\right)$ with *j* = 1, 2, 3, satisfying the equations, i.e., ${\tau_{t}}^{\left(j\right)}={t_{t}}^{\left(j\right)}+{k_{t}}^{\left(j\right)}\cdot (x_{t}+{s_{t}}^{\left(j\right)})$ = ${t_{t}}^{\left(j\right)}+{k_{t}}^{\left(j\right)}\cdot (x_{t}+r_{t}+{h_{t}}^{\left(j\right)}\cdot s)$ mod n, where *j* = 1, 2, 3. Note that winning Game 1 requires that $SA_{1}$ has never queried the oracles ExtractPartialSecret and SuperSign. Based on the above three equations, β can derive the three unknown values $x_{t},r_{t}$, and s, and outputs s as the solution of a random instance (P,Q=s⋅P) of ECDLP. So far, we have shown that β can solve the given instance of ECDLP. Next, we analyze β’s success probability $Succ_{\beta }$ of winning in Game 1.

$E_{1}$: β does not abort in all of the ExtractPartialSecret queries.
$E_{2}$: $SA_{1}$ successfully forges a valid signature $\left(ID_{t},m_{t},\sigma_{t}\right)$.
$E_{3}$: The forged signature $\left(ID_{t},m_{t},\sigma_{t}\right)$ satisfies $ID_{t}=ID^{*}$.

The corresponding probabilities of the above three events are presented. That is, $\mathrm{Pr}\left[E_{1}\right]\geq {\left(1-\frac{1}{q_{CU}+q_{H}}\right)}^{q_{EPS}}$, $\mathrm{Pr}\left[E_{2}|E_{1}\right]\geq Succ_{SA_{1}}$ and $\mathrm{Pr}\left[E_{3}|E_{1}\wedge E_{2}\right]\geq \frac{1}{q_{CU}+q_{H}}$, where $q_{CU}$, $q_{H}$ and $q_{EPS}$ are the numbers of CreateUser queries, Hash queries and ExtractPartialSecret queries. In that case, the probability of β solving the given instance of ECDLP is $Succ_{\beta }=\mathrm{Pr}\left[E_{1}\wedge E_{2}\wedge E_{3}\right]=\mathrm{Pr}\left[E_{1}\right]\mathrm{Pr}\left[E_{2}|E_{1}\right]\mathrm{Pr}\left[E_{3}|E_{1}\wedge E_{2}\right]\geq \frac{1}{q_{CU}+q_{H}}{\left(1-\frac{1}{q_{CU}+q_{H}}\right)}^{q_{EPS}}Succ_{SA_{1}}$. Clearly, β can solve ECDLP with a non-negligible probability $Succ_{\beta }$ because $Succ_{SA_{1}}$ is non-negligible. This contradicts the hardness of ECDLP. **■**

**Theorem** **2.** *The proposed certificateless signature scheme is existentially unforgeable against a super type II adversary in the random oracle model, assuming the hardness of solving ECDLP. That is, if there exists a super type II adversary* $SA_{2}$ *who can submit queries to random oracles and win in Game 2 with probability* $Succ_{SA_{2}}$, *then there is an algorithm* β *which can solve a random ECDLP instance in polynomial time with success probability* $Succ_{\beta }\geq \frac{1}{q_{CU}+q_{H}}{\left(1-\frac{1}{q_{CU}+q_{H}}\right)}^{q_{es}}Succ_{SA_{2}}$.

**Proof.**  We assume that there is a super type II adversary $SA_{2}$ breaking our proposed scheme with a non-negligible probability $Succ_{SA_{2}}$. Then we want to build a polynomial-time algorithm β which uses $SA_{2}$ to solve ECDLP. That is, β receives a random ECDLP instance $\left(P,Q=x_{t}\cdot P\right)$, with β’s goal being to derive the secret $x_{t}$. Similarly, in the Initialization phase, β picks an identity $ID^{*}$ as the challenged identity in Game 2, sets $PK_{KGC}$ and sends master key s and $params=\left(G,P,PK_{KGC},H\right)$ to $SA_{2}$. Meanwhile, β maintains two lists, i.e., $L_{H_{2}}$ and $L_{K_{2}}$. Next, in the Query phase, β can issue the following oracle queries to $SA_{2}$. Here, we skip the same oracle queries as those, i.e., CreateUser, Hash, ReplacePublicKey and SuperSign, set out in Theorem 1. In addition, β simulates other oracle queries of $SA_{2}$ as follows:

➢ $RequestPublicKey\left(ID_{t}\right)$: When $SA_{2}$ makes this query with an identity $ID_{t}$, β acts as follows:
  (1) If $ID_{t}\neq ID^{*}$, β generates two random numbers $r_{t},x_{t}\in Z_{n}^{*}$, and computes $R_{t}=r_{t}\cdot P$, $h_{t}=H\left(ID_{t},PK_{KGC}\right)$, $s_{t}=r_{t}+h_{t}\cdot s$ mod *n* and $PK_{t}=x_{t}\cdot P+R_{t}$. Then, β adds $\langle ID_{t},R_{t},h_{t}\rangle$, $\langle ID_{t},s_{t},R_{t}\rangle$ and $\langle ID_{t},PK_{t},x_{t}\rangle$ to the lists $L_{H_{1}}$ and $L_{K_{1}}$, respectively. Finally, β returns $PK_{t}$ to $SA_{2}$.
  (2) Otherwise, β selects a random value $r_{t}\in Z_{n}^{*}$, and sets $R_{t}=r_{t}\cdot P$, $h_{t}=H\left(ID_{t},PK_{KGC}\right)$, $s_{t}=r_{t}+h_{t}\cdot s$ mod *n* and $PK_{t}=x_{t}\cdot P+R_{t}$. Then, β adds $\langle ID_{t},R_{t},h_{t}\rangle$, $\langle ID_{t},s_{t},R_{t}\rangle$ and $\langle ID_{t},PK_{t},⊥\rangle$ to the lists $L_{H_{1}}$ and $L_{K_{1}}$ respectively. Finally, β returns $PK_{t}$ to $SA_{2}$.
➢ $ExtractPartialSecret\left(ID_{t}\right)$: When $SA_{2}$ makes this query with an identity $ID_{t}$, β looks for $\langle ID_{t},s_{t},R_{t}\rangle$ in $L_{K_{1}}$. If there exists a record of such a tuple, β returns $s_{t}$ to $SA_{2}$; otherwise, β makes a RequestPublicKey query with $ID_{t}$ and returns $s_{t}$ to $SA_{2}$ accordingly.
➢ $ExtractSecret\left(ID_{t}\right)$: When $SA_{2}$ makes this query with an identity $ID_{t}$, β acts as follows:
  (1) If $ID_{t}=ID^{*}$, β terminates the session.
  (2) Otherwise, β looks for $\langle ID_{t},PK_{t},x_{t}\rangle$ in $L_{K_{1}}$. If there is such a record, β returns $x_{t}$ to $SA_{2}$; otherwise, β makes a RequestPublicKey query with $ID_{t}$ and then returns $x_{t}$ to $SA_{2}$.

Finally, $SA_{2}$ outputs a forged but valid signature $\left(ID_{t},m_{t},\sigma_{t}\right)$. If $ID_{t}\neq ID^{*}$, β stops the simulation. Otherwise, β looks for $\langle ID_{t},s_{t},R_{t}\rangle$ and $\langle ID_{t},PK_{t},x_{t}\rangle$ in the list $L_{K_{1}}$. Based on the forking lemma [20], if we have the polynomial replay of β with the same random tape and different choices of hash oracle, $SA_{2}$ can further generate another signature. Eventually, we have two valid signatures, $i.e.,{\sigma_{t}}^{\left(j\right)}=\left({T_{t}}^{\left(j\right)},{\tau_{t}}^{\left(j\right)}\right)$ with *j* = 1, 2, satisfying the equations, i.e., ${\tau_{t}}^{\left(j\right)}={t_{t}}^{\left(j\right)}+{k_{t}}^{\left(j\right)}\cdot (x_{t}+{s_{t}}^{\left(j\right)})$ = ${t_{t}}^{\left(j\right)}+{k_{t}}^{\left(j\right)}\cdot (x_{t}+r_{t}+{h_{t}}^{\left(j\right)}\cdot s)$ mod *n*, where *j* = 1, 2. Note that winning Game 2 requires that the oracles ExtractSecret and SuperSign had never been queried by $SA_{2}$. With the above two linear and independent equations, β can derive the two unknown values $r_{t}$ and $x_{t}$, and outputs $x_{t}$ as the solution of the random ECDLP instance $\left(P,Q=x_{t}\cdot P\right)$. We then analyze β’s success probability $Succ_{\beta }$ of winning in Game 2. We present the events which result in β’s success:

$E_{1}$: β does not abort in all of the ExtractSecret queries.
$E_{2}$: $SA_{2}$ successfully forges a valid signature $\left(ID_{t},m_{t},\sigma_{t}\right)$.
$E_{3}$: The forged signature $\left(ID_{t},m_{t},\sigma_{t}\right)$ satisfies $ID_{t}=ID^{*}$.

The probabilities of the following equations are presented. That is, $\mathrm{Pr}\left[E_{1}\right]\geq {\left(1-\frac{1}{q_{CU}+q_{H}}\right)}^{q_{ES}}$, $\mathrm{Pr}\left[E_{2}|E_{1}\right]\geq Succ_{SA_{2}}$, and $\mathrm{Pr}\left[E_{3}|E_{1}\wedge E_{2}\right]\geq \frac{1}{q_{CU}+q_{H}}$, where $q_{CU}$, $q_{H}$, and $q_{ES}$ are the numbers of CreateUser queries, Hash queries and ExtractSecret queries. Hence, the probability of β solving the given instance of the ECDLP is $Succ_{\beta }=\mathrm{Pr}\left[E_{1}\wedge E_{2}\wedge E_{3}\right]=\mathrm{Pr}\left[E_{1}\right]\mathrm{Pr}\left[E_{2}|E_{1}\right]\mathrm{Pr}\left[E_{3}|E_{1}\wedge E_{2}\right]\geq \frac{1}{q_{CU}+q_{H}}{\left(1-\frac{1}{q_{CU}+q_{H}}\right)}^{q_{es}}Succ_{SA_{2}}$. Now, β is able to solve ECDLP with a non-negligible probability $Succ_{\beta }$ because $Succ_{SA_{2}}$ is non-negligible. This contradicts the hardness of ECDLP.  **■**

## 5. System Implementation and Performance Evaluation

To evaluate the performance of the proposed certificateless scheme, we adopt two IoT-based testbeds, i.e., Arduino Uno and Raspberry PI 2 platforms, as the major evaluation platforms in the experiments. The Arduino Uno is a microcontroller board based on the ATmega328P, i.e., an 8-bit AVR RISC-based microchip with 32 KB EEPROM and 2 KB RAM. It is a tiny platform at very low cost, and thus is suitable to evaluate the performance of IoT-based schemes. On the other hand, the Raspberry PI is a card-sized single-board computer which offers an ARM GNU/Linux kernel and 1 GB RAM and 16 GB storage. Generally speaking, the Arduino Uno platform is usually simulated as a resource-constrained device while the Raspberry PI platform is simulated as a smart object which is more powerful on computation efficiency. Hence, in our experiment the Arduino Uno is adopted as resource-constrained objects in IoT networks and the Raspberry PI 2 platform is operated as smart objects (or the mobile IoT-based gateway associated with the resource-constrained objects). The implementation environment is outlined in Table 1. It is known that current techniques for solving ECDLP need $O\left(\sqrt{n}\right)$ steps, which depend on the size of the underlying field. NIST has recommended five levels of prime fields for certain prime *n* of sizes, i.e., 192, 224, 256, 384, and 512-bit [21] with associated and recommended elliptic curves. A prime field is the field GF(n), which contains a prime number n of elements, and the security strength of which is dependent on the length of the binary expansion of n. Normally, an elliptic curve over GF(n), where $n\approx 2^{256}$, can be contrasted with finite-field cryptography (e.g., DSA) with a 3072-bit public key and a 256-bit private key, and integer factorization cryptography (e.g., RSA) with a 3072-bit value of *n*. Therefore, to strike the best balance between protocol efficiency, security robustness and system scalability, the following two conditions are considered in our system implementation.

(1) Condition (1). For the Arduino Uno, we adopt elliptic curve points over a prime field GF(n) with a 192-bit prime n, a random number generator with a 96-bit output sequence and a secure one-way hash function, i.e., SHA-3 (512-bit) [22] as the underlying crypto-modules in our proposed certificateless scheme.
(2) Condition (2). For the Raspberry PI 2 platform, the elliptic curve is with a 384-bit prime n and the random number generator is with 96-bit output sequence. In addition, SHA-3 (512-bit) is implemented as the one-way hash function.

Table 2 describes the computation cost of our proposed certificateless signature scheme implemented on the Arduino Uno with a 192-bit elliptic curve, a 96-bit random number generator and a 512-bit SHA-3, in terms of execution time of required computation components. In the pre-processing phase, we need 4.414 ms for generating four random numbers, 0.2 ms for computing $h_{i}=H\left(ID_{i},PK_{KGC}\right)$ via a SHA-3 operation with a 288-bit input sequence, 14.4 s for calculating four values $PK_{KGC}$, $R_{i}$, $s_{i}$ and $PK_{i}$ via ECC scalar multiplication operations, and 8.64 s for verifying the equation $s_{i}\cdot P=R_{i}+h_{i}\cdot PK_{KGC}$. The total computation cost of the pre-processing phase is 23.044 s. Next, during each normal operation of our proposed scheme, we require 11.537 s and 14.416 s for the sign phase and the verify phase, respectively. In the sign phase, we require 1.104 ms to generate a 96-bit $t_{i}$, and 2.88 s and 16.2 ms to compute $T_{i}$ and $k_{i}$, respectively. Note that we assume that the size of the signed message *m* is 512-bit and, thus, the input sequence of $k_{i}$ is 1408-bit. Finally, 8.64 s is needed to compute the signature value $\tau_{i}$. On the other hand, in the verify phase, we need 16.4 ms to complete the executions of $h_{i}$ and $k_{i}$, and 14.4 s to verify the equation, i.e., $\tau_{i}\cdot P=T_{i}+k_{i}\cdot \left(PK_{i}+h_{i}\cdot PK_{KGC}\right)$. Thus, we require 25.953 s in total to execute the processes of our proposed certificateless signature scheme. According to the above simulation results, we can see that the practicability of the proposed scheme is not convinced. However, in a general IoT scenario, resource-constrained sensors usually perform simple task (or command), such as the sensing and transmission of environmental parameters. This kind of data is always meaningless when it is transmitted alone. Therefore, we argue that only reasonable security density is required to guarantee basic robustness. Based on our implementation results, we find that the execution of ECC scalar multiplication operations dominates the computation cost of the proposed scheme. For better performance efficiency, we suggest that the elliptic curve points with a 64/96/160-bit prime *n*, the 64/96-bit random number generator and the SHA-3 128/256-bit can be considered during the implementation of practical applications. Table 3 shows the implementation results of the experiment with the elliptic curve with a 160-bit prime n. If we adopt the elliptic curve with a 160-bit prime n, a 96-bit random number generator and a SHA-3 with 512-bit output, around 53% of computation cost can be deducted from the case with the 192-bit elliptic curve. That is, as shown in Table 3, we only require 10.812 s, 5.421 s, and 6.771 s for executing the pre-processing phase, the sign phase, and the verify phase of our proposed scheme, respectively. It is believed that the best balance of system robustness and performance efficiency can be achieved by appropriately adjusting the system parameters of the adopted crypto-modules. Furthermore, when higher security robustness is needed, the proposed certificateless signature method can be adopted to support a key exchange (or key agreement) process and produce a session key for later secure communication via symmetric encryption (e.g., the performance of AES implementation on Arduino Uno is shown in Table 4). It is obvious that both higher security and better performance can, thus, be delivered. In brief, for resource-constrained devices, we suggest to exploit our proposed certificateless signature mechanism with 160-bit elliptic curve to construct a robust key exchange (or key agreement) process, and enjoy the performance efficiency from the symmetric encryption with an exchanged (or agreed) session key while preserving the security. Note that the same security level can be achieved via 160-bit elliptic curve and 1028-bit RSA, respectively [27].

Similarly, Table 5 describes the computation cost of our proposed certificateless signature scheme implemented on the Raspberry PI 2 platform in which the elliptic curve points is with a 384-bit prime n, the random number generator is a 96-bit output sequence, and the one-way hash function is SHA-3 (512-bit). In the pre-processing phase, 0.276 ms is required for four random number generations, 0.0051 ms is required for computing a SHA-3 operation with a 480-bit input sequence, i.e., $h_{i}=H\left(ID_{i},PK_{KGC}\right)$, 0.355 ms is required for the calculation of four values, i.e., $PK_{KGC}$, $R_{i}$, $s_{i}$ and $PK_{i}$ via ECC scalar multiplication operations, and 0.213 ms is required for verifying the equation $s_{i}\cdot P=R_{i}+h_{i}\cdot PK_{KGC}$. In total, we need 0.895 ms to execute the pre-processing phase. Next, 1.549 ms and 1.556 ms are required for executing the sign phase and the verify phase, respectively. In the sign phase, we require 1.336 ms to generate a 96-bit $t_{i}$, and to compute $T_{i}$ and $k_{i}$. Note that the input sequence of $k_{i}$ is 1792-bit. Finally, 0.213 ms is needed for computing the value $\tau_{i}$. In the verify phase, we need 1.2011 ms to compute $h_{i}$ and $k_{i}$, and 0.355 ms to verify the equation, i.e., $\tau_{i}\cdot P=T_{i}+k_{i}\cdot \left(PK_{i}+h_{i}\cdot PK_{KGC}\right)$. In brief, we require 3.105 ms in total to execute the processes of our proposed certificateless signature scheme.

Based on our implementation results, the performance bottleneck occurs at the execution of the SHA-3 hash function with a 1792-bit input sequence, i.e., about 77% (≈(1.196×2)/(1.549+1.556)) of total computation cost is dominated by this operation. Nevertheless, the computation cost of executing $h_{i}$, derived via a SHA-3 hash function with a 480-bit input sequence, is almost negligible when compared to the total computation cost. This observation inspires us to further investigate the performance evaluation of the SHA-3 hash function on the Raspberry PI 2 platform. From Table 6, we observe that the performance of SHA-3 hash function will degrade once the input sequence exceeds multiple of 576-bit, which is one of the defaulted block sizes of the SHA-3 hash function. In other words, it appears that SHA-3 hash function is more suitable for communication protocols with short messages. Normally, in a sensor-based IoT environment, communication messages operated by sensors cannot be too long, due to power consumption limitations. We, thus, argue that the proposed scheme is suitable for current IoT-based communication networks.

Based on the above results, we find that there exists one limitation in our experiment. In order to examine the practicability of the proposed scheme, the experiment adopts the Arduino Uno and Raspberry PI 2 as the evaluation platforms. However, the adopted crypto-libraries are not consistent in which Bouncy Castle Crypto APIs [23] is adopted for the Raspberry PI 2, and Fackelmann/SHA3 [24], Kmackay/micro-ecc [25] and AESLib [26] are for the Arduino Uno. In general, the evaluation platforms with different processors certainly influence the performance. On the other hand, the crypto-library may also be elegantly-tuned to fit specific processors and gain better performance efficiency. In our experiment, the Bouncy Castle Crypto APIs are generic crypto-libraries for general processors and the others (i.e., Fackelmann/SHA3 [24], Kmackay/micro-ecc [25], and AESLib [26]) are well-configured for the feasible implementation on the Arduino Uno. The performance evaluation is, thus, not under the same evaluation criteria. Fortunately, the practicability and feasibility of the proposed certificateless signature scheme is demonstrated by the experiments. Nevertheless, this limitation existed. Therefore, we suggest that this limitation can be as one of the future research directions. In addition, to pursue the best balance between the performance efficiency and security robustness, we suggest that, in the resource-constrained objects, the proposed scheme with 160-bit elliptic curve can be exploited to construct a robust key exchange process and support secure communications. Tuning the ECC crypto-module with a 192-bit (or 224/256/384/512-bit) elliptic curve to fit the resource-constrained objects is suggested as another interesting future research direction.

## 6. Related Work

In recent years, designing certificateless signature schemeswithout bilinear pairings has been extensively studied due to its effectiveness in solving the key escrow problem in identity-based cryptography, and its potential for deployment in an environment comprising resource-limited mobile devices. In this section, we first present the state-of-the-art of certificateless signature before revealing a previously unknown weakness in a recent certificateless signature mechanism proposed by Wang et al. [28]. We then present a comparative summary of our proposed scheme and relevant schemes.

### 6.1. Review of Certificateless Signature Schemes

Since Al-Riyami and Paterson [18] first proposed certificateless public key cryptography in 2003 to solve the key-escrow problem in identity-based public key cryptography, certificateless cryptography has been widely investigated for different network types. Huang et al. [17], in 2007, refined the security model presented by Al-Riyami and Paterson, and introduced type I and type II adversaries with three different power levels, namely: a normal adversary, strong adversary, and super adversary. The authors then presented a robust scheme based on bilinear pairing, and proved the security of the scheme against type I and II adversaries. Later, Gong and Li [29] introduced a provably-secure certificateless signature scheme without the use of bilinear pairing. The authors claimed that their proposed scheme is more robust than previous schemes, in terms of resilience to super type I and II adversaries. While security proof in the random oracle was presented, Yeh et al. [30,31] pointed out that the scheme is vulnerable to super type I attacker, contrary to the claims. The authors then proposed a countermeasure for the identified attacks in which the robustness against super type I and II adversaries can be guaranteed. In a latter work, Wang et al. [28] re-designed the communication procedures of the certificateless signature mechanism proposed by Yeh et al. [30,31] to enhance the computation efficiency. Specifically, costs associated with ECC-based scalar multiplication and addition operations of points are removed. However, in the next subsection, we reveal that a malicious super type I adversary can easily forge a legitimate signature to cheat any receiver as he/she wishes in this scheme.

There have been other attempts to design lightweight certificateless signcryption schemes for low-cost sensors. For example, in a 2014 work, Shi et al. [32] proposed a certificateless signcryption scheme without bilinear pairing, and proved the security of the scheme against type I and II adversaries assuming the hardness of the discrete logarithm problem. Shingh et al. [14] and Sharma et al. [15] demonstrated a RSA-based certificateless signature scheme for wireless sensor networks, which attempted to integrate RSA cryptography in certificateless signature scheme for securing resource-limited sensors. However, an exponential multiplication operation under a discrete logarithm is less efficient than point multiplication operations on elliptic curves over GF(n) under the same security level [27]. Hence, there is potential to improve the performance in the schemes reported in [14,15,32] without invalidating the security claims. Pang et al. [33] presented a bilinear pairing-based certificateless signature scheme and proved its security in the standard model. However, the proposed scheme requires significant computation due to the inherent nature of bilinear pairing. In [16], Tsai proposed a certificateless short signature scheme using bilinear pairing. It was claimed that the proposed scheme is suitable for low-bandwidth communication environment (or power-constrained devices). However, the tradeoff between communication and computation costs is not rigorously investigated, in terms of power consumption of target devices. Hence, this claim is debatable. Moreover, the current sensor-based communication environment is not as bandwidth-limited compared to a decade ago, as we have previously discussed. Therefore, we posit that computational efficiency should be prioritized over communication efficiency when designing an efficient certificateless signature scheme for IoT-based smart objects.

### 6.2. Previously Unknown Weakness in Wang et al’s (2015) CLS Scheme

We now revisit Wang et al.’s certificateless signature scheme [28], and demonstrate that the scheme is insecure against a super type I adversary.

- Revisiting the scheme:
  ➢ In the Setup phase, KGC generates a group *G* of elliptic curve points with prime order *n* and determines a generator *P* of *G*, prior to randomly selecting a master secret key $s\in Z_{p}^{*}$ and computing the master public key $PK_{KGC}=s\cdot P$. Then, KGC chooses two secure hash functions $H_{1}:{\left\{0,1\right\}}^{*}\times G\times G\to Z_{q}^{*}$ and $H_{1}:{\left\{0,1\right\}}^{*}\times {\left\{0,1\right\}}^{*}\times G\times G\times G\times G\to Z_{q}^{*}$, and publishes a set of system parameters, i.e., $params=\left(G,P,PK_{KGC},H_{1},H_{2}\right)$.
  ➢ In the PartialPrivateKeyExtract phase, given params, s and the user *i*’s identity $ID_{i}$, KGC selects a random number $r_{i}\in Z_{n}^{*}$, and computes $R_{i}=r_{i}\cdot P$, $h_{i}=H_{1}\left(ID_{i},R_{i},PK_{KGC}\right)$ and $s_{i}=r_{i}+h_{i}\cdot s$ mod *n*. Next, KGC returns the partial private key $D_{i}=\left(s_{i},R_{i}\right)$ to the user *i*. Upon receiving $D_{i}$, *i* is able to verify $D_{i}$ by examining whether two values, i.e., $s_{i}\cdot P$ and $R_{i}+h_{i}\cdot PK_{KGC}$, are identical or not since $s_{i}\cdot P=\left(r_{i}+h_{i}\cdot s\right)\cdot P=R_{i}+h_{i}\cdot PK_{KGC}$.
  ➢ In the SetSecretValue phase, given params, the user *i* randomly selects $x_{i}\in Z_{n}^{*}$ as his/her secret value.
  ➢ In the SetPublicKey phase, given params and $x_{i}$, the user *i* computes his/her public key as $PK_{i}=x_{i}\cdot P$.
  ➢ In the Sign phase, given params, $D_{i}$, $x_{i}$ and a message *m*, the user *i* selects a random value $t_{i}\in Z_{n}^{*}$, and outputs a signature $\sigma_{i}=\left(R_{i},T_{i},\tau_{i}\right)$ with a series of computed values $T_{i}=t_{i}\cdot P$, $k_{i}=H_{2}\left(ID_{i},m,T_{i},PK_{i},R_{i},PK_{KGC}\right)$ and $\tau_{i}=t_{i}+k_{i}\cdot x_{i}+s_{i}$ mod *n*.
  ➢ In the Verify phase, Given params, $ID_{i}$, $PK_{i}$, *m* and $\sigma_{i}=\left(R_{i},T_{i},\tau_{i}\right)$, the verifier computes $h_{i}=H_{1}\left(ID_{i},R_{i},PK_{KGC}\right)$ and $k_{i}=H_{2}\left(ID_{i},m,T_{i},PK_{i},R_{i},PK_{KGC}\right)$, and then checks whether the equation $\tau_{i}\cdot P=T_{i}+k_{i}\cdot PK_{i}+R_{i}+h_{i}\cdot PK_{KGC}$ holds. Note that $\sigma_{i}$ is accepted if the equation holds. That is, $\tau_{i}\cdot P=\left(t_{i}+k_{i}\cdot x_{i}+s_{i}\right)\cdot P=t_{i}\cdot P+k_{i}\cdot x_{i}\cdot P+s_{i}\cdot P=t_{i}\cdot P+k_{i}\cdot x_{i}\cdot P+\left(r_{i}+h_{i}\cdot s\right)\cdot P=T_{i}+k_{i}\cdot PK_{i}+R_{i}+h_{i}\cdot PK_{KGC}$.
- Cryptanalysis
  ➢ Suppose there exists a malicious super type I adversary *j* which seeks to forge a valid signature $\sigma_{i}=\left(R_{i},T_{i}^{'},\tau_{i}^{'}\right)$ on a message *m*' chosen by the adversary *j*. The adversary *j* eavesdrops a valid signature $\sigma_{i}=\left(R_{i},T_{i},\tau_{i}\right)$ with message *m* issued by the user *i* from any previous session, where $T_{i}=t_{i}\cdot P$, $R_{i}=r_{i}\cdot P$, $PK_{i}=x_{i}\cdot P$, $PK_{KGC}=s\cdot P$, $h_{i}=H_{1}\left(ID_{i},R_{i},PK_{KGC}\right)$, $k_{i}=H_{2}\left(ID_{i},m,T_{i},PK_{i},R_{i},PK_{KGC}\right)$, $s_{i}=r_{i}+h_{i}\cdot s$ mod *n*, and $\tau_{i}=t_{i}+k_{i}\cdot x_{i}+s_{i}$ mod *n*.
  ➢ Since the adversary *j* is a super type I adversary, *j* is able to issue an oracle query of *ExtractSecretValue*(*i*) and replace any entity’s public key including KGC’s public key. With the eavesdropped values, i.e., $T_{i}$, $R_{i}$ and $\tau_{i}$, and public values, i.e., $PK_{i}$ and $PK_{KGC}$, the adversary *j* chooses a random number $t_{a}\in Z_{n}^{*}$, and derives $T_{a}=t_{a}\cdot P$, $T_{i}^{'}=T_{a}+T_{i}$, $k_{i}^{'}=H_{2}\left(ID_{i},m^{\prime },T_{i}^{'},PK_{i},R_{i},PK_{KGC}\right)$ and $\tau_{i}^{'}=\tau_{i}-k_{i}\cdot x_{i}+k_{i}^{'}\cdot x_{i}+t_{a}=\left(t_{i}+k_{i}\cdot x_{i}+s_{i}\right)-k_{i}\cdot x_{i}+k_{i}^{'}\cdot x_{i}+t_{a}=\left(t_{i}+t_{a}\right)+k_{i}^{'}\cdot x_{i}+s_{i}$ mod *n*. Note that the secret $x_{i}$ is retrieved via *ExtractSecretValue*(*i*) oracle query.
  ➢ So far, the adversary *j* can forge a valid signature $\sigma_{i}^{'}=(R_{i},T_{i}^{'},\tau_{i}^{'})$ on the chosen message *m*'. It is obvious that the equation $\tau_{i}^{'}\cdot P=\left[\left(t_{i}+t_{a}\right)+k_{i}^{'}\cdot x_{i}+s_{i}\right]\cdot P=\left(t_{i}+t_{a}\right)\cdot P+k_{i}^{'}\cdot x_{i}\cdot P+\left(r_{i}+h_{i}\cdot s\right)\cdot P=\left(T_{a}+T_{i}\right)+k_{i}^{'}\cdot PK_{i}+r_{i}\cdot P+h_{i}\cdot s\cdot P=T_{i}^{'}+k_{i}^{'}\cdot PK_{i}+R_{i}+h_{i}\cdot PK_{KGC}$ holds. Therefore, the resistance to signature forgery attack cannot be guaranteed under the assumption of existing a malicious super type I adversary.

### 6.3. Security and Performance Comparative Summary

We now benchmark the security and performance of the proposed certificateless signature with those of Gong and Li [29], Wang et al. [28] and Tsai [16]. From Table 7, we observe that our proposed scheme and Tsai’s scheme [16] enjoy the same security level–resilience to super type I and II adversaries. However, Gong and Li’s scheme [29] still suffers from vulnerability to signature forgery attack via super type I adversary [30] as does Wang et al.’s scheme [28], as presented in Section 6.2.

A comparative summary of performance efficiency is presented in Table 8, where the evaluation metrics are of the inverse operation (*T_inv_*), bilinear pairing operation (*T_bp_*), ECC-based scalar multiplication operation for points (*T_em_*), ECC-based addition operation for points (*T_eadd_*), multiplication operation (*T_m_*), addition operation (*T_add_*), one-way hash function (*T_h_*), and random number generator operation (*T_g_*). It is clear that our proposed scheme outperforms Gong and Li’s scheme [29] and Wang et al.’s scheme [28] by eliminating the computation costs of (1*T_m_*, 1*T_h_*, 2*T_eadd_*, 2*T_h_*) and (1*T_eadd_*), respectively. When compared to Tsai’s scheme [16], the tradeoff between the computation cost (1*T_m_*, 1*T_add_*, 1*T_h_*) and (1*T_inv_*, 2*T_bp_*) is observed. It is clear that bilinear pairing operation is more inefficient than ECC point-based operations, i.e., scalar multiplication and addition. Hence, we can claim that our proposed scheme is more efficient and practical than Tsai’s scheme [16] with a better performance efficiency.

## 7. Conclusions

In this paper, we presented a new certificateless signature scheme for IoT-based smart objects. We proved the security of the proposed scheme against the super type I and II adversaries, as well as demonstrating the utility of the scheme in IoT-oriented testbeds. For passive objects with constrained computation ability and limited power capability, we argued that the proposed certificateless signature scheme with 160-bit elliptic curve can be exploited to construct a key exchange (or key agreement) process with a reasonable security robustness. Around 5.421 s and 6.771 s are required for performing the sign phase and the verify phase of our proposed scheme, respectively. For active objects with powerful computation efficiency, we suggested considering the proposed certificateless signature scheme with at least 384-bit elliptic curve and SHA-3 (512-bit) to pursue the highest security due the affordability of computation cost on the Raspberry PI platform. Findings from the implementation showed that low computation cost, i.e., 1.549 ms and 1.556 ms, is required to perform the execution processes of the sign phase and verify phase, respectively. Moreover, we compared the security and performance of our scheme with those of Gong and Li [29], Wang et al. [28] and Tsai [16], as well as revealing a previously unknown vulnerability in Wang et al.’s scheme [28] (where a malicious super type I adversary can easily forge a valid signature on any message and cheat receivers at will).

## Figures and Tables

**Table 1:** Implementation environment.

Environment	Description
Arduino Uno	Atmel ATmega328P 8-Bit 16MHz AVR Architecture Memory 2 KB RAM/32 KB EEPROM
Raspberry PI 2	Broadcom BCM2836 @ 1 GHz Quad-Core ARM Cortex-A7 Architecture with 1 GB DDR2 RAM and SanDisk 16 GB Class 10 SD Card
Programming Language	(For Raspberry PI 2) Eclipse 3.8 with Oracle Java 8 ARM (For Arduino Uno) ANSI C
Crypto API	(For Raspberry PI 2) The Bouncy Castle Crypto APIs [23] (For Arduino Uno) Fackelmann/SHA3 [24], Kmackay/micro-ecc [25], AESLib [26]

**Table 2:** The computation cost of our proposed certificateless signature scheme implemented on the Arduino Uno with Condition (1).

Phase	Computation Cost	Execution Time	Total
Pre-processing	Generate s, $r_{i}$, $x_{i}$, $ID_{i}$ (96-bit)	4.414 ms	23.044 s
	Compute $h_{i}$ (SHA-3 with 288 bit input sequence)	0.2 ms	
	Compute $PK_{KGC}$, $R_{i}$, $s_{i}$, $PK_{i}$ (ECC 192-bit)	14.4 s	
	Verify $s_{i}\cdot P=R_{i}+h_{i}\cdot PK_{KGC}$ (ECC 192-bit)	8.64 s	
Sign	Generate $t_{i}$ (96-bit)	1.104 ms	11.537 s
	Compute $k_{i}$ (SHA-3 with 1408-bit input sequence) ^1^	16.2 ms	
	Compute $T_{i}$ (ECC with 192-bit)	2.88 s	
	Compute $\tau_{i}=t_{i}+k_{i}\cdot (x_{i}+s_{i})$ (ECC 192-bit)	8.64 s	
Verify	Compute $h_{i}$ (SHA-3 with 288-bit input sequence)	0.2 ms	14.416 s
	Compute $k_{i}$ (SHA-3 with 1408-bit input sequence) ^1^	16.2 ms	
	Verify $\tau_{i}\cdot P=T_{i}+k_{i}\cdot \left(PK_{i}+h_{i}\cdot PK_{KGC}\right)$ (ECC 192-bit)	14.4 s	

^1^ Suppose the size of message *m* is 512-bit.

**Table 3:** The computation cost of our proposed signature scheme implemented on the Arduino Uno with a 160-bit elliptic curve, a 96-bit random number generator, and a 512-bit SHA-3.

Phases of the Proposed Scheme	Total Execution Time
Pre-processing phase	10.812 s
Sign phase	5.421 s
Verify phase	6.771 s

**Table 4:** The computation cost of AES implemented on the Arduino Uno.

Input Sequence of AES	Encryption/Decryption
AES-128 with 32/64/128/256 Bytes Input Sequence	0.63 ms
AES-256 with 32/64/128/256 Bytes Input Sequence	0.87 ms

**Table 5:** The computation cost of our proposed certificateless signature scheme implemented on the Raspberry PI 2 with Condition (2).

Phase	Computation Cost	Execution Time	Total
Pre-processing	Generate s, $r_{i}$, $x_{i}$, $ID_{i}$ (96-bit)	0.276 ms	0.895 ms
	Compute $h_{i}$ (SHA-3 with 480-bit input sequence)	0.0051 ms	
	Compute $PK_{KGC}$, $R_{i}$, $s_{i}$, $PK_{i}$ (ECC 384-bit)	0.355 ms	
	Verify $s_{i}\cdot P=R_{i}+h_{i}\cdot PK_{KGC}$ (ECC 384-bit)	0.213 ms	
Sign	Generate $t_{i}$ (96-bit)	0.069 ms	1.549 ms
	Compute $k_{i}$ (SHA-3 with 1792-bit input sequence) ^1^	1.196 ms	
	Compute $T_{i}$ (ECC with 384-bit)	0.071 ms	
	Compute $\tau_{i}=t_{i}+k_{i}\cdot (x_{i}+s_{i})$ (ECC 384-bit)	0.213 ms	
Verify	Compute $h_{i}$ (SHA-3 with 480-bit input sequence)	0.0051 ms	1.556 ms
	Compute $k_{i}$ (SHA-3 with 1792-bit input sequence) ^1^	1.196 ms	
	Verify $\tau_{i}\cdot P=T_{i}+k_{i}\cdot \left(PK_{i}+h_{i}\cdot PK_{KGC}\right)$ (ECC 384-bit)	0.355 ms	

^1^ Suppose the size of message *m* is 512-bit.

**Table 6:** The computation cost of SHA-3 with different length input sequences on Raspberry PI 2.

SHA-3 Operation	Execution Time
SHA-3 with 576-bit input sequence	0.412 ms
SHA-3 with 1152-bit input sequence	0.939 ms
SHA-3 with 1728-bit input sequence	1.194 ms
SHA-3 with 2304-bit input sequence	1.726 ms
SHA-3 with 2880-bit input sequence	2.260 ms
SHA-3 with 3456-bit input sequence	2.407 ms
SHA-3 with 4032-bit input sequence	2.807 ms
SHA-3 with 4608-bit input sequence	3.215 ms
SHA-3 with 5184-bit input sequence	4.084 ms
SHA-3 with 5760-bit input sequence	4.430 ms

**Table 7:** A comparative summary: security.

	Gong & Li’s Scheme [29]	Wang et al’s Scheme [28]	Tsai’s Scheme [16]	Our proposed Scheme
Resistance to Super Type I Adversary	No	No	Yes	Yes
Resistance to Super Type II Adversary	Yes	Yes	Yes	Yes

**Table 8:** A comparative summary: performance.

	Sign Phase	Verify Phase	In Total
Gong & Li’s scheme [29]	1*T_em_* + 2*T_m_* + 2*T_add_* + 2*T_h_* + 1*T_g_*	4*T_em_* + 3*T_eadd_* + 3*T_h_*	5*T_em_* + 2*T_m_* + 3*T_eadd_* + 2*T_add_* + 5*T_h_* + 1*T_g_*
Wang et al’s scheme [28]	1*T_em_* + 1*T_m_* + 2*T_add_* + 1*T_h_* + 1*T_g_*	3*T_em_* + 3*T_eadd_* + 2*T_h_*	4*T_em_* + 1*T_m_* + 3*T_eadd_* + 2*T_add_* + 3*T_h_* + 1*T_g_*
Tsai’s scheme [16]	1*T_inv_* + 1*T_em_* + 1*T_m_* + 1*T_add_* + 1*T_h_*	2*T_bp_* + 2*T_em_* + 2*T_eadd_* + 2*T_h_*	1*T_inv_* + 2*T_bp_* + 3*T_em_* + 1*T_m_* + 2*T_eadd_* + 1*T_add_* + 3*T_h_*
Our proposed scheme	1*T_em_* + 1*T_m_* + 2*T_add_* + 1*T_h_* + 1*T_g_*	3*T_em_* + 2*T_eadd_* + 2*T_h_*	4*T_em_* + 1*T_m_* + 2*T_eadd_* + 2*T_add_* + 3*T_h_* + 1*T_g_*
